# Incidence rate and malignancy risk in new nodules in a lung cancer screening programme

**DOI:** 10.1007/s00330-025-11576-3

**Published:** 2025-04-24

**Authors:** Andrew W. Creamer, Carolyn Horst, Priyam Verghese, Ruth Prendecki, Amyn Bhamani, Helen Hall, Jennifer L. Dickson, Sophie Tisi, Chuen Ryan Khaw, John McCabe, Kylie Gyertson, Anne-Marie Hacker, Laura Farrelly, Allan Hackshaw, Arjun Nair, Anand Devaraj, Sam M. Janes, the SUMMIT consortium, Chuen Ryan Khaw, Kylie Gyertson, Arjun Nair, Sam M. Janes, Jennifer L. Dickson, Carolyn Horst, Sophie Tisi, Helen Hall, Priyam Verghese, Andrew Creamer, Thomas Callender, Ruth Prendecki, Amyn Bhamani, Mamta Ruparel, Allan Hackshaw, Laura Farrelly, Jon Teague, Anne-Marie Mullin, Kitty Chan, Rachael Sarpong, Malavika Suresh, Samantha L. Quaife, Anand Devaraj, Vicky Bowyer, Ethaar El-Emir, Judy Airebamen, Alice Cotton, Kaylene Phua, Elodie Murali, Simranjit Mehta, Janine Zylstra, Karen Parry-Billings, Columbus Ife, April Neville, Paul Robinson, Laura Green, Zahra Hanif, Helen Kiconco, Ricardo McEwen, Dominique Arancon, Nicholas Beech, Derya Ovayolu, Christine Hosein, Sylvia Patricia Enes, Qin April Neville, Jane Rowlands, Aashna Samson, Urja Patel, Fahmida Hoque, Hina Pervez, Sofia Nnorom, Moksud Miah, Julian McKee, Mark Clark, Jeannie Eng, Fanta Bojang, Claire Levermore, Anant Patel, Sara Lock, Rajesh Banka, Angshu Bhowmik, Ugo Ekeowa, Zaheer Mangera, William M. Ricketts, Neal Navani, Terry O’Shaughnessy, Charlotte Cash, Magali Taylor, Samanjit Hare, Tunku Aziz, Stephen Ellis, Anthony Edey, Graham Robinson, Alberto Villanueva, Hasti Robbie, Elena Stefan, Charlie Sayer, Nick Screaton, Navinah Nundlall, Lyndsey Gallagher, Andrew Crossingham, Thea Buchan, Tanita Limani, Kate Gowers, Kate Davies, John McCabe, Joseph Jacob, Karen Sennett, Tania Anastasiadis, Andrew Perugia, James Rusius

**Affiliations:** 1 Lungs For Living, UCL Respiratory, University College London, London, UK; 2 University College London Hospitals NHS Foundation Trust, London, UK; 3 Cancer Research UK and UCL Cancer Trials Centre, University College London, London, UK; 4 National Heart and Lung Institute, Imperial College, London, UK; 5 Royal Brompton and Harefield NHS Foundation Trust, London, UK; 6 Centre for Prevention, Detection and Diagnosis, Wolfson Institute of Population Health, Barts and The London School of Medicine and Dentistry, Queen Mary University of London, London, UK; 7 Royal Free London NHS Foundation Trust, London, UK; 8 Whittington Health NHS Trust, London, UK; 9 Barking, Havering and Redbridge University Hospitals NHS Trust, Essex, UK; 10 Homerton University Hospital Foundation Trust, London, UK; 11 The Princess Alexandra Hospital NHS Trust, Essex, UK; 12 North Middlesex University Hospital NHS Trust, London, UK; 13 Barts Health NHS Trust, London, UK; 14 North Bristol NHS Trust, Bristol, UK; 15 Royal United Hospitals Bath NHS Foundation Trust, Bath, UK; 16 Surrey and Sussex Healthcare NHS Trust, Surrey, UK; 17 King’s College Hospital NHS Foundation Trust, London, UK; 18 The Princess Alexandra Hospital NHS Trust, London, UK; 19 University Hospitals Sussex NHS Foundation Trust, Sussex, UK; 20 Royal Papworth Hospital NHS Foundation Trust, Cambridge, UK; 21 Centre for Medical Image Computing (CMIC), London, UK; 22 Killick Street Health Centre, London, UK; 23 Tower Hamlets Clinical Commissioning Group, London, UK; 24 Noclor Research Support, London, UK

**Keywords:** Lung cancer, Cancer screening, Pulmonary nodules (solitary), Pulmonary nodules (multiple)

## Abstract

**Introduction:**

There is limited evidence for the malignancy risk posed by new nodules appearing at annual screening rounds or at short-term interval nodule follow-up (NFU) CTs in lung cancer screening programmes. We investigated incidence rate and malignancy risk in new nodules appearing at NFU and at first annual CT in a screening cohort and investigated nodule and participant characteristics which predicted malignancy.

**Methods:**

11,566 participants underwent baseline CT screening between April 2019 and April 2020. CTs were read in conjunction with computer-aided detection software with semi-automated volumetry. Nodule management was based on British Thoracic Society guidelines, with the addition of a lower threshold for new solid nodules appearing at incident rounds; those ≥ 30 and < 200 mm^3^ underwent a further 3-month interval scan, and new nodules ≥ 200 mm^3^ were referred directly for definitive investigation.

**Results:**

New nodules were identified in 8.4% of participants at NFU-CT and 11.1% at Y1. 0.63% (95% confidence interval (CI) 0.016–3.433) of new nodules at NFU-CT and 2.98% (95% CI 1.83–4.57) at annual CT proved malignant. Malignancy risk in new nodules at Y1 was 1.67% in nodules < 30 mm^3^, 2.2% in nodules 30–200 mm^3^ and 11.0% in nodules > 200 mm^3^. No nodules with typical perifissural or subsolid morphology were malignant. There was no significant difference in age, smoking status, smoking history or predicted cancer risk between participants with new nodules which proved malignant and those which were benign.

**Conclusion:**

Our findings validate the need for lower volume thresholds for further surveillance or definitive investigation in new solid nodules at annual scans. Malignancy risk in new nodules with subsolid or typical perifissural morphology and in new nodules appearing in a shorter time frame of NFU CTs is low.

**Key Points:**

***Question*** *What is the incidence and malignancy risk of new nodules appearing at annual and nodule follow-up interval CTs in lung cancer screening?*

***Findings*** *New nodules were seen in 11.1% and 8.4% of participants at annual low-dose CT and 3-month interval CT, respectively. Malignancy risk at annual CT increased with nodule size.*

***Clinical relevance*** *In a lung cancer screening programme, new nodules at annual and nodule follow-up CTs occur in around 1 in 10 participants. Lower size thresholds for further surveillance or definitive investigation should be considered compared to nodules at baseline CT.*

**Graphical Abstract:**

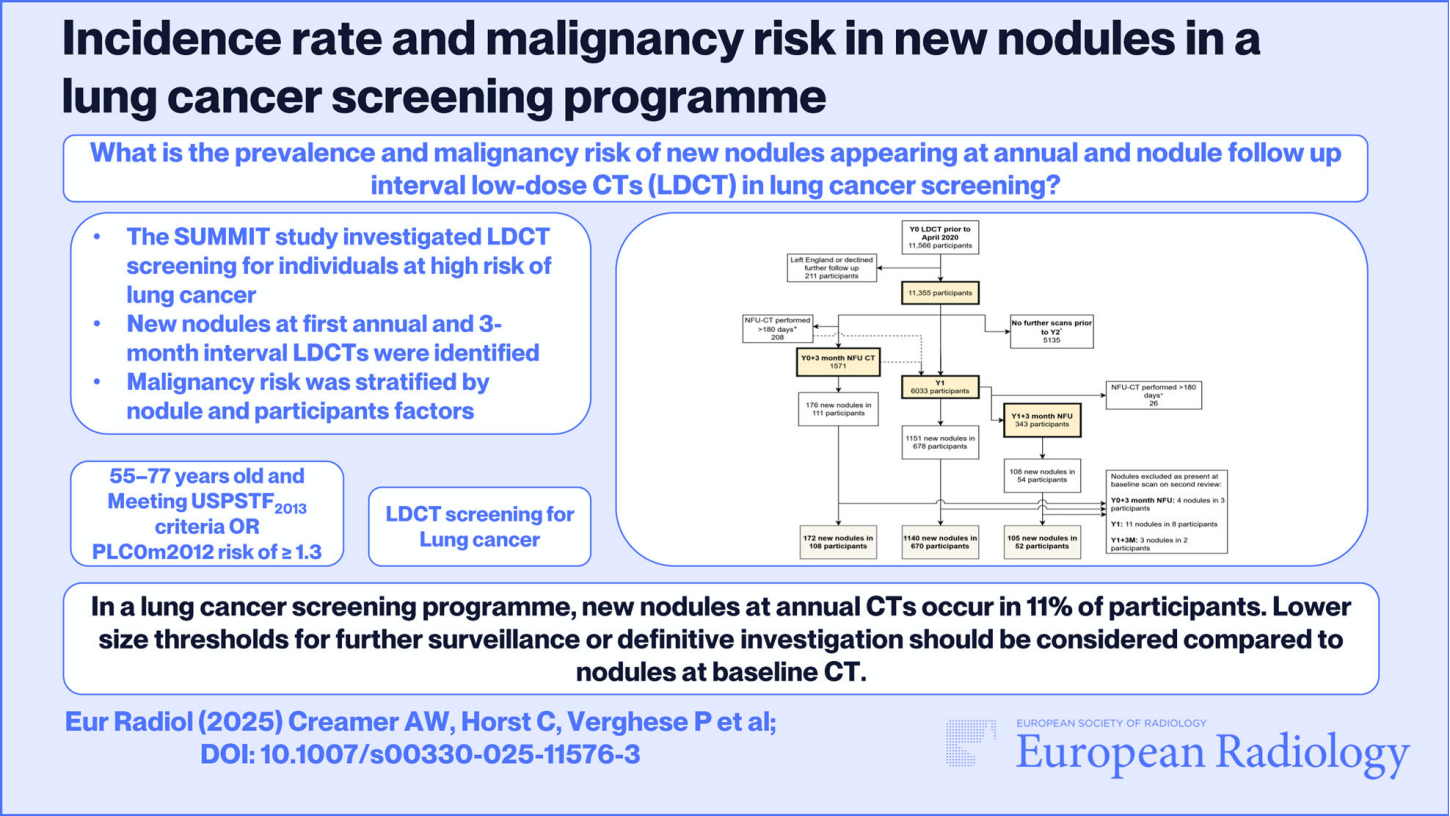

## Background

There is a substantial body of evidence investigating malignancy risk in lung nodules identified at baseline CT in lung cancer screening (LCS) programmes [1–4]. However, understanding of the risk of malignancy posed by new nodules seen at subsequent scans is more limited. As lung cancer screening becomes established as an annual/biennial process, positive scans will increasingly be driven by the development of new nodules, for which currently validated baseline risk prediction models (e.g., the Brock score) are not applicable. An evidence-based approach to these nodules is therefore essential for effective screening programmes.

As well as appearing at annual/biennial screening rounds, new nodules can also appear on nodule follow-up (NFU) CTs after a shorter interval, such as CTs acquired 3 months after identification of an indeterminate nodule [5, 6]. Such a finding may itself lead to a further 3-month interval scan, potentially doubling, tripling, or even quadrupling the number of scans a participant undergoes with each year of screening. It has been hypothesised that nodules developing within a short time period have a high likelihood of being benign or inflammatory by virtue of their very rapid growth rate [5, 7]. However, the malignancy risk in this group of nodules has not been widely studied in the setting of lung cancer screening, so there is, at present, no evidence to support more or less frequent surveillance.

Evidence from the NELSON lung cancer screening trial [8] found that the probability of a new solid nodule ≥ 27 mm^3^ at subsequent screening round (1–2 years after baseline) representing cancer was higher than that of an equivalently sized nodule identified on the baseline scan, and therefore advocated a lower size threshold for surveillance or referral for investigation for new nodules identified at annual or biennial screening rounds. LungRADS advises lower size thresholds for the management of new nodules (advising 6-month and 3-month NFU-CT for new solid nodules < 6 mm diameter (< 113 mm^3^) and 6 to < 8 mm (113 to < 268 mm^3^), respectively, and definitive investigation in new solid nodules ≥ 8 mm (268 mm^3^)), but no distinction is made regarding whether the new nodule develops at an annual screening visit or in the shorter timeframe of a nodule follow-up scan. Fleischner Society [9] (for incidentally detected) and British Thoracic Society [5] (for incidentally- and screen-detected nodules) guidelines make no specific recommendations for new nodules identified at 3-month follow-up CTs.

The aims of this analysis were first to investigate the frequency and malignancy risk of new nodules identified at annual screens and at 3-month follow-up CTs and second to identify nodule and patient features that may predict malignancy risk in new solid nodules.

## Methods

### Study design and participants

The SUMMIT Study is a prospective observational cohort study which aims to assess the implementation of Low-Dose Computed Tomography (LDCT) screening and validate a multi-cancer early detection blood test (NCT03934866). In brief, participants were 55–77 years old, met the US Preventive Services Task Force 2013 screening criteria [10] or had a PLC0_m2012_ risk of ≥ 1.3% [11] and attended three annual lung health checks (baseline (Y0), year 1 (Y1), and year 2 (Y2)). LDCT was performed for all participants at Y0 and Y2, with participants with a negative Y0 scan randomly allocated to LDCT or no LDCT at Y1 in a 1:1 ratio (hence the number of LDCT scans performed at Y1 was expected to be significantly lower than that performed at Y0). Results from the multi-cancer early detection blood test were not available to clinicians managing the screening programme and are not considered in this analysis.

### Procedures

LDCTs were performed on GE Revolution multi-slice scanners at maximal inspiration in one continuous craniocaudal acquisition without intravenous contrast. Thin collimation (0.625 mm slice thickness) volumetric images were reconstructed using a soft tissue algorithm and 50% ASIR-V iterative reconstruction. Images were analysed with computer-aided detection (CADe) software for semi-automated volume measurements (Veolity, MeVIS). All images were subsequently reviewed by an experienced thoracic radiologist, with access to the CADe results. Individual nodules were tracked across serial CT scans. We did not have a minimum size threshold for recording nodules, but reporting radiologists were instructed to record all CADe-detected nodules but not to identify micronodules below the threshold for CADe detection (< 4 mm) [12].

Nodule management was based on British Thoracic Society Guidelines [5] and has been described previously [13]. In brief, SUMMIT uses a volumetric approach to solid nodules. Solid nodules at baseline scan with a volume ≥ 300 mm^3^ (or ≥ 8 mm diameter when unreliable segmentation cannot be achieved) with a Brock score of ≥ 10% at baseline or at a subsequent scan were referred for definitive investigation. Solid nodules of ≥ 80 and < 300 mm^3^ on baseline scan underwent nodule follow-up (NFU) CT at 3 months. Nodules stable at 3-month interval scan underwent further scans at Year 1 and Year 2. Nodules demonstrating growth with a volume of > 200 mm^3^ were referred to the MDT for definitive assessment. Drawing on results from the NELSON study [8], specific additional guidelines for new nodules were implemented: new solid nodules ≥ 30 mm^3^ and < 200 mm^3^ underwent a further 3-month interval scan, and new nodules ≥ 200 mm^3^ were referred directly for definitive investigation.

Subsolid nodules were assessed using the total long-axis diameter and solid core diameter, where relevant. Pure ground glass nodules underwent annual surveillance, whilst subsolid nodules underwent interval scan at 3 months. Referral for definitive assessment was advised where the solid component was ≥ 8 mm diameter or there was any changing morphology or growth of the solid component or for pure ground glass nodules where total diameter exceeded 30 mm.

Subsequent investigations for nodules referred from screening were at the discretion of the multidisciplinary team (MDT) but typically included PET-CT and/or nodule biopsy.

Participants with negative baseline scans were randomly allocated to scan or no scan at Y1 in a 1:1 ratio and therefore only a proportion of participants underwent LDCT at Y1. All participants were invited for LDCT at Y2.

New nodules were identified by the reporting radiologist. Radiologists were allowed to ignore new nodularity that they deemed unequivocally inflammatory (such as tree-in-bud or clustered nodularity). To ensure only genuinely new nodules were evaluated in this analysis, nodules first reported at NFU or Y1 CT but considered visible in retrospect by the reporting radiologist on the Y0 LDCT were not included in this analysis. Furthermore, as an additional step to ensure any malignant nodule was indeed new, the prior study scans for all nodules that proved malignant underwent a second review by the senior study radiologist (A.N.) to confirm they were not present on prior scans.

To ensure our data was representative of a typical 3-month NFU scenario, participants who had an interval of longer than 180 days before the NFU CT were excluded (delays were primarily due to the SARS-CoV-2 pandemic).

### Outcomes

Cancer was confirmed by histology or diagnosed clinico-radiologically by MDT assessment, where biopsy/surgical procedures were felt to be inappropriate. Cancers were linked to individual screen-detected nodules based on anatomical location, with subsequent imaging (CT-guided biopsies or CT-PET) reviewed where required. Nodules were recorded as benign either based upon histology following MDT referral, radiological resolution or if size stability or shrinkage based on volumetry was demonstrated over at least 12 months. All participants were followed up by national cancer registries (NCRAS) to identify cancers diagnosed outside of the study. Where participants did not complete follow-up within the study, benignity was assumed based on an absence of cancer diagnosis in the registry within the follow-up period (median follow-up after Y0 + 3M 35.8 months (IQR 30.2–38.6), after Y1 25.7 months (IQR 21.6–27.7), after Y1 + 3M NFU 22.3 months (IQR 19.3–25.7)).

Stage was determined by the TNM (tumour, node, metastasis) classification of malignant tumours, 8th edition [14]. We included pulmonary metastases from extrapulmonary cancers where new pulmonary nodules were the first presentation of the cancer, and have specified these.

### Statistical analysis

We investigated features that may predict malignancy in new nodules, including nodule type, volumetric size and rate of growth for solid nodules, and the participants’ underlying risk of lung cancer (assessed by age, smoking history, current smoking status, history of malignancy, and with the PLCO_m2012_ composite risk score [11]). To evaluate the rate of growth of new solid nodules, a virtual volume doubling time (vVDT) was calculated by:

$${{{\rm{Virtual}}}}\; {{{\rm{Volume}}}}\; {{{\rm{Doubling}}}}\; {{{\rm{Time}}}}=[{{\rm{ln}}}2{\,{{{\rm{x}}}}\,}\Delta {{{\rm{T}}}}]/{{{\rm{ln}}}}[{{{\rm{V}}}}_{{{\rm{new}}}}/{{{{\rm{V}}}}}_{0}]$$

Where V_new_ is nodule volume at first appearance and ΔT the time (days) between baseline scan and the scan on which the nodule first appeared. V_0_ was set at 4mm^3^, the volume (to the nearest whole number) of a spherical nodule of 2 mm diameter. While CT resolution can visualise nodules smaller than this, we acknowledged that for readers to reliably distinguish nodules against a background of anatomical structures—especially on low-dose CT—a larger threshold was required.

Cancer risk was defined as the number of participants with new nodules that proved malignant divided by the total number of participants with new nodules of that type. Where participants had more than one new nodule, they were represented by the single largest new nodule, ensuring a 1:1:1 ratio of individuals:nodules:cancers in the data. Continuous variables were assessed by Welch’s T-test (parametric data) and the Mann–Whitney U test (nonparametric) with data presented as medians and overall range. Categorical variables were compared with Fisher’s exact test. All tests were two-sided, and significance was defined as *p*-value < 0.05. Statistical analysis was performed with R (version 4).

This study analyses outcomes from all participants who had a baseline scan between study commencement (4 April 2019) and temporary closure for the SARS-CoV-2 pandemic on 18 March 2020 (*N* = 11,566).

## Results

**Fig. 1:**
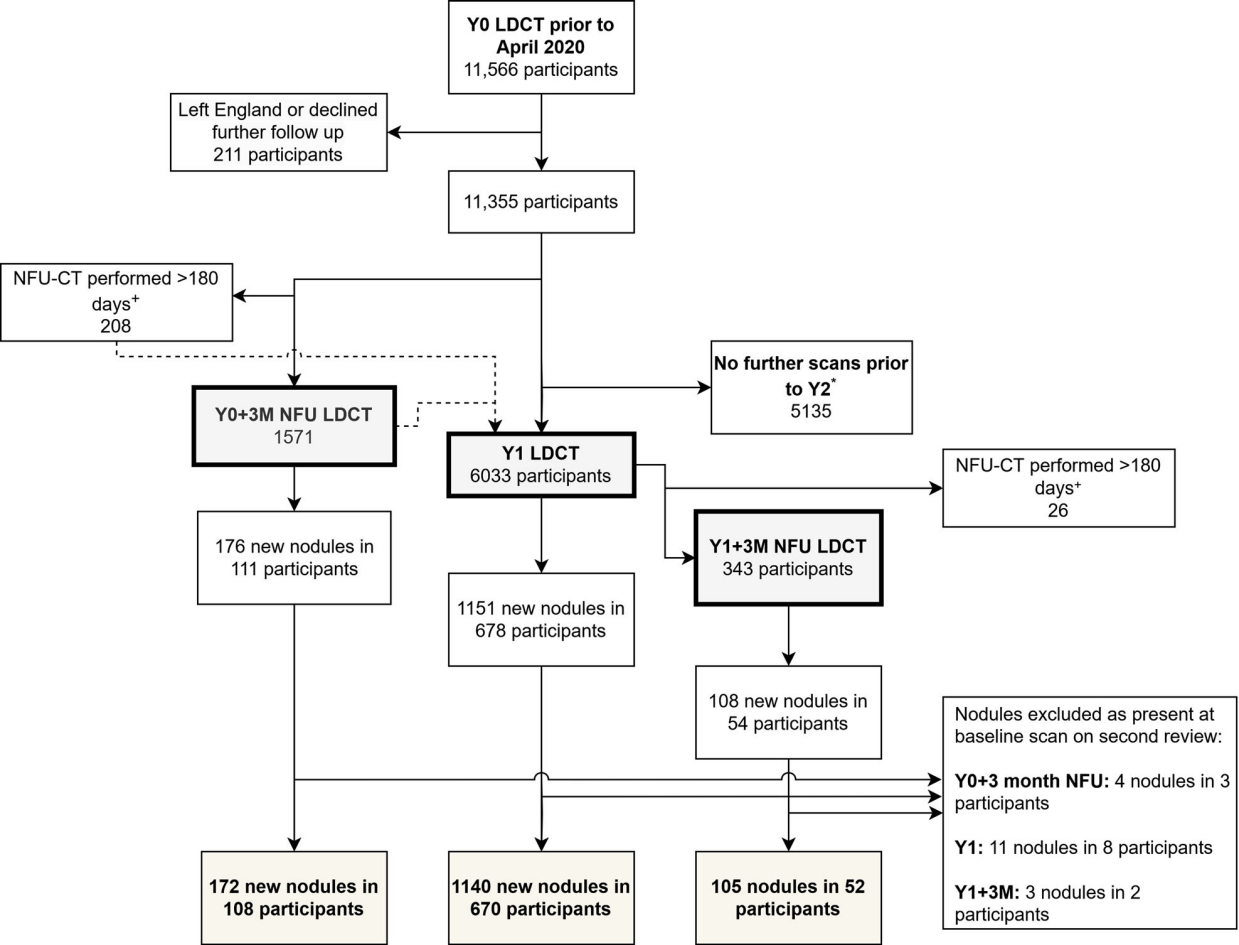
Participants undergoing low-dose CT at baseline, Y0 + 3-month nodule follow-up, Year 1 and Y1 + 3-month nodule follow-up timepoints. LDCT, low-dose CT; NFU-CT, nodule follow-up CT; Y0, year 0 (baseline); Y1, year 1. ^+^ To ensure the population of new nodules appearing at NFU-CT were representative of those appearing in the 3-month timeframe, participants where NFU-CT scans were performed > 180 days after Y0/Y1 were not included in the NFU-CT analysis. The reason for significant delays was primarily due to disruption from the SARS-CoV-2 pandemic. ^‡^ Second review was limited to nodules reported as new which proved malignant. * Participants with negative baseline LDCTs were randomised to scan or no scan at Y1 in a 1:1 ratio, therefore only a proportion of participants who had baseline LDCT underwent a further scan at Y1

Genuinely new nodules were reported in 670 of the 6033 participants who had a Y1 LDCT (11.1%) (Fig. 1). Of these, 339 (5.6%) had new solid nodule(s), 112 (1.8%) had new indeterminate nodular consolidation, 65 (1%) had new pure ground glass (pGGN) nodule(s) and 178 (3.0%) had new nodule(s) with typical perifissural morphology (Some participants had more than one new nodule identified).

New nodules were reported in 108 (6.9%) of the 1571 participants and 52 (15.2%) of the 343 participants who had a Y0 + 3-month and Y1 + 3-month NFU CT, respectively. Taking these time points together (160 participants with new nodules in 1914 NFU LDCTs, 8.4%), 88 (4.6%) had new solid nodules, 40 (2.1%) had new indeterminate nodular consolidation, 17 (0.9%) had new pGGN and 25 (1.3%) had new perifissural nodules (Table 1).

**Table 1:** Incidence rate of cancer in new nodules by nodule type

Nodule type	Y0 + 3M NFU	Y1 + 3M NFU	NFU CTs combined	Y1
Total ppts with ≥ 1 new nodule/total ppts scanned	108/1571 (6.9%)	52/343 (15.2%)	160/1914 (8.4%)	670/6033 (11.1%)
No. of cancers	0	1	1	20
No. of cancers/nodule type (*n*, %)				
Solid	0/52 (0.0%)	1/36 (2.3%)	1/88 (1.1%)	19/339 (5.6%)
Part-solid	0/5 (0.0%)	0/2 (0.0%)	0/7 (0.0%)	0/35 (0.0%)
Pure ground glass	0/14 (0.0%)	0/3 (0.0%)	0/17 (0.0%)	0/65 (0.0%)
Perifissural	0/20 (0.0%)	0/5 (0.0%)	0/25 (0.0%)	0/178 (0.0%)
Consolidation	0/25 (0.0%)	0/15 (0.0%)	0/40 (0.0%)	1/112 (0.8%)

Data is presented at a per-participant level, with each participant with one or more nodules of that type represented in the row (As some participants had multiple new nodules of different types, the sum of the rows by nodule types exceeds that of the total number of participants with new nodules)

*Y1* year 1 CT, *Y0/Y1 + 3M NFU* nodule follow-up after baseline or year 1 annual CT, *ppts* participants

When the NFU-CTs after Y0 and Y1 (Y0+3M and Y1+3M) were combined, the incident frequency for new nodules was lower at NFU CTs than at Y1 (8.4% vs 11.1%, relative risk (RR) 0.75, *p* = 0.0006). The proportion of these new nodules meeting criteria for further assessment (i.e., further CT or direct MDT referral) was also lower (3.1% vs 5.6%, RR 0.55, *p* < 0.0001).

New nodules in 21 participants were subsequently confirmed as cancer (Table 1). Crude risk of malignancy in new nodules was therefore 3.0% (20/670, 95% CI 1.83–4.57) at annual scans and 0.6% (1/160, 95% CI 0.016–3.433) at NFU scans (OR 4.76, 95% CI 0.75–198.7, *p* = 0.1524). Ten out of 21 (48%) cancers diagnosed from new nodules were squamous cell carcinomas, 5 (24%) were adenocarcinomas, one (5%) was an adenosquamous carcinoma and two (9.6%) were small cell lung cancers. 3 (14%) malignant nodules were metastases from non-lung primaries (stomach, colon and breast). 62% (13/21) were diagnosed at stage IA (Table 2).

**Table 2:** Stage and histological type of cancers diagnosed in new nodules at nodule follow-up and annual CT

	Timepoint nodule first identified at	
	Nodule follow-up CT	Annual CT
Total	1	20
Histological type		
Small cell lung cancer	0	1
Squamous cell carcinoma	1	10
Adenocarcinoma	0	4
Metastases from non-lung primary	0	3
Adenosquamous	0	1
Non-small cell lung cancer not otherwise specified	0	1
Stage		
IA	0	13
1B	1	0
2B	0	1
3A	0	1
3B	0	1
4	0	4

We investigated whether nodule features predicted malignancy in new nodules at annual and NFU LDCTs. No malignancy was found in any new nodules with subsolid morphology (*n* = 124) or typical perifissural morphology (*n* = 203) regardless of time point. Nodules with these morphologies were excluded from further volume/rate of growth analyses.

Malignancy risk in new solid nodules according to size thresholds is shown in Table 3. Analysing new nodules at NFU and annual CT scans together, sensitivity for malignancy with a 30 mm^3^ threshold was 90.5% (95% CI 69.6–98.8%) and specificity 28.7% (95% CI 24.88–32.78). With an 80 mm^3^ threshold, sensitivity was 81.0% (95% CI 58.09–94.55%) and specificity 53.6% (49.25–57.94).

**Table 3:** Volume and malignancy risk in new solid nodules at NFU and Y1 scans (combined)

	At NFU CT		At Y1		Total	
	Malignant nodules/total nodules	Lung cancer probability (95% CI)	Malignant nodules/total nodules	Lung cancer probability (95% CI)	Malignant nodules/total nodules	Lung cancer probability (95% CI)
Total	1/120	0.83% (0.02–4.56)	20/427	4.68% (2.88–7.14)	21/547	3.84% (2.39–5.81)
Volume (mm^3^)						
0–30	0/33	0% (0.00–10.58)	2/120*	1.67% (0.20–5.89)*	2/153	1.31% (0.16–4.64)
30–80	0/34	0% (0.00–10.28)	2/99	2.02% (0.25–7.11)	2/133	1.50% (0.18–5.33)
80–200	0/15	0% (0.00–21.80)	2/81	2.47% (0.30–8.64)	2/96	2.08% (0.25–7.32)
> 200	1/38	2.70% (0.07–14.16)	14/127	11.02% (6.16–17.80)	15/165	9.09% (5.18–14.55)
Virtual VDT						
< 25 days	1/57	1.75% (0.04–9.39)	0/0	NA	1/57	1.75% (0.04–9.39)
25–50 days	0/40	0.0% (0.00–8.81)	4/41	9.76% (2.72–23.13)	4/81	4.94% (1.36–12.16)
< 50 days	1/97	1.03% (0.03–5.61)	4/41	9.76% (2.72–23.13)	5/138	3.62% (1.19–8.25)
50–100 days	0/13	0.0% (0.00–24.71)	11/168	6.55% (3.31–11.41)	11/181	6.08% (3.07–10.61)
> 100 days	0/10	0.0% (0.00–30.85)	5/218	2.29% (7.49–5.27)	5/228	2.19% (0.72–5.04)

Data is presented at a per-participant level. Analysis excludes those of subsolid or typical perifissural morphology. Where participants had more than one new nodule, they are represented by the new nodule of the largest volume

*VDT* volume doubling time, *95% CI* 95% confidence interval

* As CADe limit is < 4 mm (equivalent to 33 mm^3^), new nodules smaller than this were not consistently detected or reported. Two malignant nodules were seen to be new at Y1 with size < 30 mm^3^ in retrospect after growing at the Y2 scan. As new benign micronodules (which did not subsequently grow) would not have been consistently reported, the true denominator is likely higher, and hence risk lower, for new nodules <30 mm^3^

Virtual VDT (vVDT) had wide confidence intervals, with a trend toward an inverse-U shaped relationship with malignancy risk: 1.75% (95% CI 0.04–9.39) of new nodules with a vVDT of < 25 days, 4.94% (95% CI 1.36–12.16) of nodules with vVDT of 25–50 days, 3.62% (95% CI 3.07–10.61) of nodule with a vVDT of 50–100 days and 2.19% (95% CI 0.72–5.04) of new nodules with vVDT > 100 days proving malignant (Table 3).

Finally, we investigated participant-level features which may predict malignancy (Table 4). There was no significant difference in age, smoking history, current smoking status or history of malignancy between participants with new malignant nodules and those with new benign nodules. There was no significant association of malignancy risk of new nodules with PLCO_m2012_ on univariate or multivariate analysis (OR 1.042, *p* = 0.171, adjOR 1.043, *p* = 0.171).

**Table 4:** Nodule and participant characteristics in new benign and malignant solid nodules at NFU and Y1 scans

	Malignant new nodules	Benign new nodules	*p*-value
Total	21	526	
Nodule factors:			
Median volume (mm^3^)	384.3 (108.0–1133.6)	65.4 (25.2–253.7)	0.3554
Median virtual VDT (days)	62.6 (50.7–96.1)	79.3 (46.4–131.1)	0.8270
Participant factors:			
Median age	69.0 (67.0–72.0)	66.0 (62.0–72.0)	0.0633
Current smoker (percentage)	57.1%	54.5%	0.9847
Mean smoking history (Pack years)	20 (15.0–20.0)	20.0 (15.0–25.0)	0.9224
Mean PLCO_m2012_	5.655 (3.927–10.486)	4.082 (2.163–7.109)	0.1272

Analysis includes nodules with nodular consolidation morphology but excludes those of typical perissural morphology. Where participants had more than one new nodule, they are represented by the nodule of the largest volume. Numerical values are given as median with intraquartile range in parenthesis

## Discussion

We present data from one of the largest studies of malignancy risk in new nodules at annual CT in lung cancer screening to date [8, 15] and the first (to our knowledge) to investigate risk in new nodules at short-term surveillance CT. We found that (1) the probability of lung cancer in new nodules arising at annual screening CT is overall small, but is not negligible (3.0%); (2) lung cancer risk is very low for new nodules emerging within the short interval of a 3-month follow-up CT (0.63%); (3) lower size thresholds for surveillance (30 mm^3^) and immediate referral (200 mm^3^) of new nodules at 1 year from baseline CT advocated in previous studies [8] is reinforced.

Despite the increasing adoption of LCS as an annual or biennial process in the US [10], UK [16] and other countries, there remains a paucity of evidence to guide management decisions for new nodules. Our data add to previous studies showing that this is not a negligible concern. Our annual incident nodule rate of 11.1% (of which 5.6% met the criteria for further surveillance or MDT assessment) is somewhat higher than the 5–7% reported in NELSON [8] and 7.5% in PLuSS [17]. When extrapolated to population-level screening, this incident rate represents a substantial clinical problem that requires an evidence-based management approach.

In new nodules appearing at annual CT scans, our data provides further validation of findings from the NELSON study that a lower threshold for further surveillance or investigation is required. Compared to data from NELSON [8] for new nodules at annual scans, we identified a malignancy risk in new solid nodules at Y1 of < 30 mm^3^ of 1.7% (compared to 0.5% in new nodules < 27 mm^3^ in NELSON), a risk of 2.2% in new nodules 30–200 mm^3^ (vs 3.1% in 27–206 mm^3^), and 11.0% in nodules > 200 mm^3^ (vs 16.9% in ≥ 206 mm^3^). Our reported risk in new nodules < 30 mm^3^ is slightly higher than that reported in the NELSON study, which likely represents the fact that as our CADe detection threshold (and hence which nodules radiologists were instructed to report) was < 4 mm (equivalent to ~33 mm^3^), nodules smaller than this may not be detected or reported. The true denominator for this size threshold is therefore likely higher and the overall risk lower. By contrast, we report numerically lower cancer probabilities in new nodules > 30 mm^3^, which may represent different definitions of ‘new’ nodules—we excluded those cases where a malignant nodule could be seen in retrospect on baseline scan, whilst NELSON used a reporting threshold of 15 mm^3^ (equivalent to 3 mm diameter) so may have included malignant nodules present at baseline CT of < 15 mm^3^ which subsequently grew. Nevertheless, our results provide prospective validation in a separate cohort for the use of lower size thresholds for new nodules at annual CT scans, corresponding to 30–80 mm^3^ for intermediate risk requiring surveillance, and > 200 mm^3^ requiring referral for definitive assessment.

Beyond nodule size, morphology can also be used to guide management decisions. Consistent with previous findings from the NELSON study [18], we found new nodules of typical perifissural morphology had a 0% risk of malignancy. We therefore propose that, as at baseline scans [5], they can be disregarded. We also found a 0% risk of malignancy in new subsolid nodules, supporting previous findings that the majority of these lesions seen on a single scan will resolve spontaneously [19]. We did not find patient characteristics (age, smoking history, PLCO_m2012_) helpful in distinguishing malignant from benign nodules in this context. We explored whether calculating vVDT could help distinguish new benign and malignant nodules. As only one cancer was diagnosed in nodules appearing in the shorter timeframe of an NFU-CT, the confidence intervals were wide. We observed that all but one malignant new nodule had vVDTs of 45 days or longer, suggesting that nodules growing faster than this are likely to represent a benign inflammatory process, consistent with previous findings [7]. However, further research in new nodules appearing after a wider range of time intervals is required to assess whether this metric is of additional utility in predicting malignancy beyond volume and morphology.

New nodules appearing within the shorter timeframe of NFU scans warrant specific consideration. Although less frequent than at annual scans, these nodules have an incidence rate of 8.4% and are therefore a sufficiently common scenario to require an evidence-based management approach. Our findings show that truly new nodules appearing within a 3–6-month period are highly unlikely to be malignant, with a crude risk of 0.63%. There are however caveats to this. First, close attention must be paid to baseline scans to look for evidence of a growing nodule. On second review, five out of six malignant nodules labelled as ‘new’ at NFU scans could be seen in retrospect at the prior baseline/annual scan. In this scenario, persistence (with growth) across serial scans, rather than volume at first appearance is likely to be the factor most predictive of malignancy. Second, this analysis was limited to nodule follow-up scans performed at 3–6 months—scans delayed beyond this are likely to more closely represent the risk profile seen at annual scans. The question of whether further surveillance is ever indicated for such new nodules appearing at short-term follow-up scans is worth examination. The low malignancy risk of 0.6% is equivalent to that of baseline solid nodules of < 100 mm^3^ (0.7%) reported in NELSON that led to BTS [5] and European Position statement [20] guidelines advising that such nodules do not require follow-up. The potential benefit of a diagnosis made after a further 3-month interval scan (rather than at 9 months at the next annual screen) must be weighed against the costs of further follow-up scans (both financial and in anxiety for the participant). For new nodules appearing at NFU scans, we would first advocate a close review of the preceding scan; previous research from the SUMMIT cohort has found evidence that clear growth of a previously small nodule is suspicious for malignancy [21]. For truly new nodules, further imaging is likely to depend on the individual programme, but delaying surveillance to the next screening visit may be a justifiable approach.

The histological type and stage of cancers presenting as new nodules at interval LDCT provide further insight into the significance of this group. Although the majority (13/20) of the 20 cancers detected as new nodules at annual CT were diagnosed at stage IA, four were diagnosed at stage 4, of which 3 were non-lung malignancies presenting with lung metastases. This serves as a reminder for those reporting screening CTs to be alert to the possibility of new nodules being the presenting feature of extrapulmonary malignancies.

Research requiring nodule-level outcomes in large screening cohorts is challenging, and limitations to this analysis should be acknowledged. Whilst the vast majority of patients underwent further imaging within or outside the study, only one further annual CT was performed in SUMMIT. While this was sufficient to confirm resolution of the majority of benign nodules or demonstrate clear growth in malignant ones, it may underestimate the malignancy of adenocarcinoma-spectrum lesions presenting as evolving subsolid nodules, for which we followed BTS guidelines of conservative management with referral for further surveillance. Nevertheless, as the primary question of this analysis is whether new nodules should be managed differently at the time they are first identified, we do not think this significantly alters the findings of our paper. Furthermore, we mitigated this by using national cancer registry data to ensure cancers diagnosed outside the study (within the follow-up timeframe) were also included. As discussed previously, a further limitation is the variable recording of very small (< 4 mm diameter) nodules. The CADe limit for nodule detection of < 4 mm (equivalent to 33 mm^3^) meant new nodules smaller than this were not consistently detected or reported, but two malignant nodules were seen (in retrospect) to be new at Y1 of size < 30 mm^3^ after growing at the Y2 scan. As new benign micronodules (which did not subsequently grow) would not have been consistently reported, the true number of new nodules < 30 mm^3^ is likely higher, and hence malignancy risk is lower for new nodules of this size. Such a limitation is difficult to avoid (inter-observer variability in identifying such small nodules is recognised in addition to the limits of CADe detection [22]), but an awareness of this facilitates interpretation of our findings.

In conclusion, our results validate the need for lower size thresholds for further surveillance or definitive investigation for new nodules appearing at annual screening rounds. In 124 and 203 participants with new nodules with subsolid and typical perifissural morphology respectively, we found malignancy risk in both these groups to be zero. Lastly, we found malignancy risk in truly new nodules appearing between 3–6 months in NFU screening rounds to be less than 1%.

The findings will serve to develop an evidence base for guiding management decisions in these scenarios.

## Abbreviations

CADe: Computer-aided detection
CI: Confidence interval
LDCT: Low-dose CT
LCS: Lung cancer screening
MDT: Multidisciplinary team
NFU CT: Nodule follow-up CT
PLCO_m2012_: Prostate, Lung, Colorectal and Ovarian 2012 prediction model
vVDT: Virtual volume doubling time
VDT: Volume doubling time
Y0: Year 0 (baseline screen)
Y1: Year 1 (first annual screen)
